# Modulatory Effects of *Tetraselmis chuii* Gastrointestinal Digests on Human Colonic Microbiota

**DOI:** 10.3390/foods14122106

**Published:** 2025-06-16

**Authors:** Marta Majchrzak, Samuel Paterson, Javier Gutiérrez-Corral, Dulcenombre Gómez-Garre, Adriana Ortega-Hernández, Miguel Ángel de la Fuente, Blanca Hernández-Ledesma, Pilar Gómez-Cortés

**Affiliations:** 1 Institute of Food Science Research (CIAL, CSIC-UAM), Nicolás Cabrera 9, 28049 Madrid, Spain; marta.majchrzak@csic.es (M.M.); samuel.paterson@csic.es (S.P.); mafl@if.csic.es (M.Á.d.l.F.); 2 Department of Applied Physical Chemistry, Autonoma University of Madrid, Francisco Tomás and Valiente 7, 28049 Madrid, Spain; 3 Laboratory of Infectious Diseases, Hospital Clínico San Carlos-Instituto de Investigación Sanitaria San Carlos (IdISSC), C/Prof. Martín Lagos, 28040 Madrid, Spain; jgutierrezcorral@salud.madrid.org; 4 Microbiota and Vascular Biology Laboratory, Hospital Clínico San Carlos-Instituto de Investigación Sanitaria San Carlos (IdISSC), C/Prof. Martín Lagos, 28040 Madrid, Spain; mgomezgarre@salud.madrid.org (D.G.-G.); a.ortega.hernandez@hotmail.com (A.O.-H.); 5 Biomedical Research Networking Center in Cardiovascular Diseases (CIBERCV), Av. Monforte de Lemos, 3-5, 28029 Madrid, Spain; 6 Faculty of Medicine, Universidad Complutense de Madrid (UCM), Plaza Ramón y Cajal, 28040 Madrid, Spain

**Keywords:** *Tetraselmis chuii*, microalgae, in vitro digestion, colonic fermentation, butyric acid, microflora, short chain fatty acids

## Abstract

*Tetraselmis chuii* is a microalga commercialized because of its richness in health-beneficial molecules. Previous studies have profusely demonstrated the biological properties of compounds isolated from *T. chuii*, but data are not yet available on the impact that gastrointestinal digestion could exert. This article describes the passage of *T. chuii* through the gastrointestinal tract, combining the INFOGEST procedure and in vitro colonic fermentation to examine potential effects on the human colonic microflora composition and its metabolic activity. Microbial plate counting was conducted to determine the different groups of microorganisms. Amplification of the 16S ribosomal RNA gene was performed via polymerase chain reaction to examine in detail the main genera of bacteria, and its metabolic activity was evaluated by measuring of short-chain fatty acids (SCFAs) by gas chromatography. The presence of *T. chuii* modified the fecal microbiota. Although the evolution of lactic acid bacteria and *Enterococcus* spp. content during 72 h showed that the use of *T. chuii,* compared to fructopolysaccharides such as inulin, would not provide nutritional advantages, the microalgae extract contributed to a significant decrease in *Clostridium, Staphylococcus,* and *Enterobacteriaceae*. Furthermore, *T. chuii* increased the relative abundance of *Akkermansia* and *Butyricimonas*, genera considered highly beneficial. In correlation with the presence of these microorganisms, the results show that the presence of *T. chuii* favored the release of SCFA, such as acetic (20 mM), propionic (>5 mM), isovaleric (0.3 mM), isobutyric (0.15 mM), and, mainly, butyric (>2 mM), after 72 h colonic fermentation, being indicators of gut health. These findings suggest that *T. chuii* has potential as a functional ingredient for promoting health through its modulatory effects on the intestinal microbiota.

## 1. Introduction

Microalgae have received attention as a promising source of nutrients, being a sustainable production alternative compared to traditional foods [1,2]. Dried biomass of *Tetraselmis chuii* has been authorized in the European Union as a food supplement [3], and phytoplankton lyophilized from this microalga was also approved for novel food use in Canada [4] and the USA [5]. Furthermore, the Scientific Committee of the *Spanish Agency for Food Safety and Nutrition* has reported that the consumption of *T. chuii* as an ingredient does not generate harmful effects on human health [6]. This species has a high nutritional value and is of considerable interest for the biotechnological production of useful compounds, e.g., essential fatty acids, antioxidants (carotenoids and phenolic compounds), starch and bulk lipids, and oils [3].

In rat models, different doses of *T. chuii* freeze-dried biomass did not adversely affect the growth rate, clinical signs, blood parameters, organ weights, or histopathology [7]. Furthermore, long-term supplementation with TetraSOD^®^ (a functional ingredient consisting solely of lyophilized *T. chuii* powder) reduced oxidative stress and inflammatory disturbances linked to metabolic syndrome [8]. Its beneficial effects against this metabolic disorder included the promotion of endogenous antioxidant defense mechanisms in the liver, the modulation of oxidative stress and inflammatory markers in plasma, as well as the positive modulation of genes involved in antioxidant, anti-inflammatory, and immune pathways in different organs. In human cell lines, Cokdinleyen et al. [9] highlighted the neuroprotective potential of extracts of this microalga.

Since its authorization as a food ingredient, the use of *T. chuii* in nutraceutical production for humans is growing worldwide, conferring on microalgae the ability to generate an extensive range of relevant physiological effects. *T. chuii* supplementation elicited improvements in ergospirometric and haematological values in football players [10], and sustained performance as well as lowered muscle damage across repeated exercise bouts [11]. This ingredient appeared to operate through an elevating oxidative capacity in skeletal muscle. Furthermore, García et al. [12] reported that daily supplementation with this microalgae extract (TetraSOD^®^) could improve hematological, hormonal, biochemical, and anthropometric parameters in healthy individuals. However, most of the metabolic mechanisms underlying the effects of *T. chuii* remained unknown, and more research was claimed to clarify the pathways of action of this microalga in the human body.

It is well known that the different species of the genus *Tetraselmis* are an excellent source of a diversity of active compounds, including lipids, proteins, carbohydrates, and other minor molecules, such as polyphenols, carotenoids, or vitamins [13]. The presence of some of these molecules in microalgae biomass could be associated with modulatory effects on intestinal microorganisms [14,15]. The health benefits of prebiotics metabolism by human gut microbiota encompass the improvement of gastrointestinal function and barrier homeostasis, an increase in mineral absorption, modulation of energy metabolism and satiety, as well as reduction in the risk of intestinal infections [14]. However, despite its importance, the effects of *T. chuii* consumption on the gastrointestinal function, along with their impact on the human gut microbiota, have not been monitored, and there is still a lack of information regarding its digestibility and bioaccessibility. To help fill this void, this article examined, for the first time, the passage of *T. chuii* through the gastrointestinal tract, combining simulated gastrointestinal digestion based on the INFOGEST protocol [16] with in vitro colonic fermentation and to evaluate potential effects on the human colonic microbiota composition and its metabolic functionality.

## 2. Materials and Methods

### 2.1. Biomass and Reagents

*T. chuii*-lyophilized biomass was donated from AlgaEnergy S.A. (Madrid, Spain). The total lipid content (34.57 ± 2.89 g/100 g biomass), protein content (32.19 ± 0.29 g/100 g biomass), and carbohydrate content (7.58 ± 0.28 g/100 g biomass) of the specific *T. chuii*-lyophilized biomass used in this work was described in our previous work [17]. Pepsin (EC 232-629-3; 3200 units/mg protein), pancreatin (232-468-9; 8X USP), potassium persulfate (K_2_S_2_O_8_), monosodium phosphate (NaH_2_PO_4_), disodium phosphate (Na_2_HPO_4_), monopotassium phosphate (KH_2_PO_4_), calcium chloride (CaCl_2_), hydrochloric acid (HCl), sodium hydroxide (NaOH), sodium chloride (NaCl), yeast extract, K_2_HPO_4_ NaHCO_3_, MgSO_4_·7H_2_O, CaCl_2_·6H_2_O, Tween-80, hemin, vitamin K, L-cysteine, phosphoric acid (H_3_PO_4_), and inulin from chicory (food grade) were purchased from Sigma-Aldrich (St. Louis, MO, USA).

### 2.2. INFOGEST Gastrointestinal Digestion

Following the INFOGEST protocol described by Brodkorb et al. [16], simulated gastrointestinal digestion was performed. The oral phase involved dissolving 1 g of dried *T. chuii* biomass in distilled water, adding human saliva, and incubating the mixture at 37 °C with 130 rpm agitation for 2 min (Environmental Shaker-Incubator ES 20/60, Biosan Medical-biological Research & Technologies, Warren, MI, USA).

After completing the oral phase, simulated gastric fluid including CaCl_2_ solution and pepsin (ratio enzyme:substrate, E:S, 1:100, *w*/*w*) was added, adjusting the pH to 3.0. The gastric phase was conducted at 37 °C for 2 h. The orogastric digest was mixed with simulated intestinal fluid and the pH was adjusted to 7.0. Then, CaCl_2_ solution, pancreatin (E:S 1:3, *w*/*w*), and bile (1:50, *w*/*w*) were added. After 2 h at 37 °C, the enzymes were inactivated by heating at 95 °C for 5 min in a Memmert thermostatized bath (Schwabach, Germany). The final digest was centrifuged at 2000× *g* for 30 min at 4 °C in an EppendorfTM Centrifuge 5804R (Hamburg, Germany). Five digestion replicates were conducted, and the precipitates corresponding to the non-absorbable fractions (NAFs) were pooled and stored at −20 °C until the colonic fermentation assays were carried out. Next, 1 g of inulin, a well-known fermentable natural carbohydrate with positive prebiotic effects on host intestinal health and previously used as a reference to compare the effects of other microalgae species on gut microbiota [18,19], was also digested in quintuplicate and used as a control.

### 2.3. Static Colonic Fermentation and Microbial Plate Counting

The fecal inoculum was prepared by combining 1 g of feces obtained from a healthy volunteer who maintained a balanced and diverse diet, free of any dietary limitations, and exhibited a stool consistency corresponding to type 4 on the Bristol Stool Scale [20], with 10 mL of PBS solution (pH 7.0), and homogenized using a Stomacher 400 Circulator (Seward, AK, USA). The fermentation flasks were prepared following the protocol of Tamargo et al. [21], using 6 mL of the fecal inoculum and 3 g of the NAF obtained after *T. chuii* INFOGEST digestion. Incubations with inulin were also carried out as a control. All fermentation flasks were incubated for 72 h and 120 rpm to simulate the environmental conditions of the distal region of the human large intestine (pH 6.8, 37 °C, and anaerobic atmosphere). Aliquots were taken at 0, 24, 48, and 72 h for microbial counts.

Ten-fold serial dilutions of each colonic sample were plated on different types of media, as Jiménez-Arroyo et al. [22] described. Plate counting was conducted in triplicate, and data were expressed as log colony-forming units (CFUs)/mL. Aliquots of 2 mL were centrifuged at 10,000 rpm for 10 min at 4 °C; the supernatants were filtered and stored at −20 °C for short-chain fatty acids (SCFAs) and ammonium analysis. Pellets were stored at −80 °C for metagenomic analysis.

### 2.4. Extraction and Quantification of Microbial DNA

Microbial DNA was isolated and extracted from the precipitates obtained at 0 h and 48 h fermentation using the QIAamp Fast DNA Stool mini kit (Quiagen, Hilden, Germany). Shortly, 100 mg of stool samples was resuspended in 1 mL InhibitEX Buffer, vigorously mixed, and incubated at 95 °C for 5 min. Then, samples were centrifuged at 22,000 g, leaving DNA in the supernatant. Next, 200 μL DNA supernatant was mixed with 15 μL Proteinake K and 200 μL Buffer AL, and incubated at 70 °C for 10 min. Subsequently, 200 μL ethanol was added, mixed by vortexing, and 600 μL lysate was applied to the QIAamp spin column, which was centrifugated at 22,000 g for 1 min. The QIAamp spin column was placed in a new tube, and 500 μL Buffe AW1 was added and centrifugated at 22,000 g for 1 min. This step was repeated, adding 500 μL Buffer AW2 and centrifuging for 3 min. The QIAamp spin column was transferred to a new tube, and 200 μL Buffer ATE was applied. After incubating 1 min at room temperature, the column was centrifugated at 22,000 g for 1 min to elute DNA. DNA integrity was assessed with the BioAnalyzer 2100 (Agilent, Santa Clara, CA, USA), and the concentration was quantified with the fluorimeter Qubit 3.0 using the dsDNA HS assay (Life Technologies S.A., Alcobendas, Madrid, Spain).

### 2.5. 16S Ribosomal RNA Gene Sequencing

The 16S rRNA gene was sequenced as previously reported [23]. Briefly, for each sample, two reactions (one per primer set) were prepared, each containing 15 µL of 2X Master Mix, 3 µL of 10X 16S Primer Set, and 5 ng of DNA template using the Ion 16S Metagenomics Kit (Life Technologies S.A.). Then, the tubes were placed in a thermal cycler under the following conditions: 95 °C for 10 min, 25 cycles of 95 °C for 30 s, 58 °C for 30 s, 72 °C for 20 s, and a final extension at 72 °C for 7 min. After purification of the PCR products, the amplicon ends were repaired by combining 79 µL of pooled amplicons (80 ng), 20 µL of 5X End Repair Buffer, and 1 µL of End Repair Enzyme in a 1.5 mL Eppendorf^®^ LoBind^®^ tube, resulting in a final volume of 100 µL. The mixture was vigorously mixed by pipetting and incubated at room temperature for 20 min. The DNA was then purified and eluted in 25 µL of low TE buffer. For attaching of molecular identifiers, a ligation and nick repair reaction was prepared in a 0.2 mL PCR tube by combining 25 µL of purified DNA with 10 µL of 10X Ligase Buffer, 2 µL of Ion P1 Adapter, 2 µL of Ion Xpress™ Barcode, 2 µL of dNTP Mix, 49 µL of nuclease-free water, 2 µL of DNA Ligase, and 8 µL of Nick Repair Polymerase, using the Ion Express Barcode Kit Adapters (Life Technologies S.A.). The mixture was vigorously mixed and placed in a thermal cycler under the following conditions: 25 °C for 15 min, 72 °C for 5 min, and a final hold at 4 °C. Libraries were then amplified using the Ion Plus Fragment Library Kit (Life Technologies S.A.) by adding 5 µL of low TE to approximately 20 µL of the purified, adapter-ligated library. A PCR reaction was prepared by mixing 100 µL of Platinum™ PCR SuperMix High Fidelity, 5 µL of Library Amplification Primer Mix, and 25 µL of unamplified library, for a total of 130 µL. The mix was divided equally into two 0.2 mL PCR tubes, and subjected to PCR amplification with the following program: 95 °C for 5 min, 6 cycles of 95 °C for 15 s, 58 °C for 15 s, and 70 °C for 1 min. A final hold at 4 °C was maintained for up to 1 h. Finally, the contents of the two tubes were pooled into a single 1.5 mL Eppendorf LoBind^®^ tube, and the amplified libraries were purified and eluted in 20 µL of Low TE buffer. Then, libraries were subjected to clonal amplification by emulsion PCR using the Ion OneTouchTM 2 System (Thermo Fisher Scientific, Waltham, MA, USA), and each sample was sequenced with the Ion S5^TM^ System with an Ion 520^TM^ Chip (Life Technologies S.A.).

### 2.6. Bioinformatics Analysis

Bioinformatic analysis was conducted using in-house pipelines. Following filtering, the files were converted to FASTQ files using BamTools [24]. Subsequently, the primers were removed and the reads were split by 16 regions (V2, V3, V4, V5, V6-7, V8, V9) using Split_On_Primers.py. Reverse reads were reverse complement, and every read was trimmed at 165 pb. RESCRIPT was used to format the last available Silva 16S database (v138.2). The QIIME2 (version 2024.2) pipeline was used to dereplicate for each of the 16 regions, remove the singletons, group sequences at 99% identity to the Operational Taxonomic Unit (OTU), and align to the Silva 16S database (v138.2). Finally, chimeras, mitochondria, and chloroplasts were removed using vsearch. To create a single OTU table of abundances, each hypervariable region per sample was added together. To mitigate the different sequencing depth impact on alpha (α)-diversity and beta (β)-diversity measurements, each sample was rarefied to the minimum number of sequences between samples. QIIME2 was used to explore α-diversity metrics, assessing Shannon and Pielou’s evenness indexes. In addition, QIIME2 was also used to evaluate the diversity similarity across samples, calculating the Bray Curtis dissimilarity index. Statistical differences were analyzed using the PERMANOVA test with 999 permutations. The Silva 16S reference database associates a specific taxon with each OTU; as a result, it can be obtained the relative abundance table per taxonomic level. To identify features associated with microalgae ingestion, the phylum and genus relative abundances were compared among groups. The Python package matplotlib was used to create the plots.

### 2.7. Chemical Analysis

The fermentative activity of the colonic microbiota was assessed by determining the SCFA profile. Analysis was carried out by gas chromatography according to García-Villalba et al. [25]. A gas chromatograph (Agilent, Santa Clara, CA, USA) equipped with a DB-WAX capillary column (30 m, 0.325 mm internal diameter × 0.25 μm thickness, Agilent) was used. After injection, the column was held at 50 °C for a 2 min injection and a temperature-programmed ramp at 15 °C/min to 150 °C. Then, it was followed by two new ramps of 5 °C/min up to 200 °C and 15 °C/min to 240 °C. Finally, the temperature was held at 240 °C for 20 min. Helium was the carrier gas with a column inlet pressure set at 1.5 mL/min. The injector and the flame ionization detector temperatures were 250 °C and 260 °C, respectively. Quantitative data were obtained by calculating the area of each compound relative to the internal standard (2-methylvaleric acid). Analyses were carried out in triplicate.

The proteolytic activity of colonic microbiota was evaluated by determining the protein content and the production of ammonium ion (NH_4_^+^) in the supernatants obtained after colonic fermentation. The protein concentration of each sample was determined by the bicinchoninic acid method, using the Pierce BCA kit (Thermo Fisher Scientific, Waltham, MA, USA), as previously described [26]. Bovine serum albumin was used as a standard. The NH_4_^+^ production was determined using the Photometric Spectroquant^®^ ammonium reagent test (Merck & Co., Kenilworth, NJ, USA), measuring the absorbance at 690 nm with a Biotek SynergyTM HT plate reader (Winooski, VT, USA). The results were expressed as mg NH_4_^+/^L. Both analyses were carried out in triplicate.

### 2.8. Statistical Analysis

The data of plate counting, SCFAs, protein, and ammonium content are expressed as the mean ± standard error mean (SEM). Differences among groups were analyzed with a one-way ANOVA followed by a Tukey test, a Kruskal–Wallis test, or a paired *t*-test as appropriate. The ANCOM-BC2 v.2.8.1 R package was used to perform log-fold change analysis at the genus level, accounting for paired samples. Genera with more than 0.3% relative abundance in at least one group (Inulin or *T. chuii*) were included.

## 3. Results and Discussion

### 3.1. Effects of T. chuii on the Intestinal Bacterial Populations During Colonic Fermentation: Plate Counting

**Figure 1:**
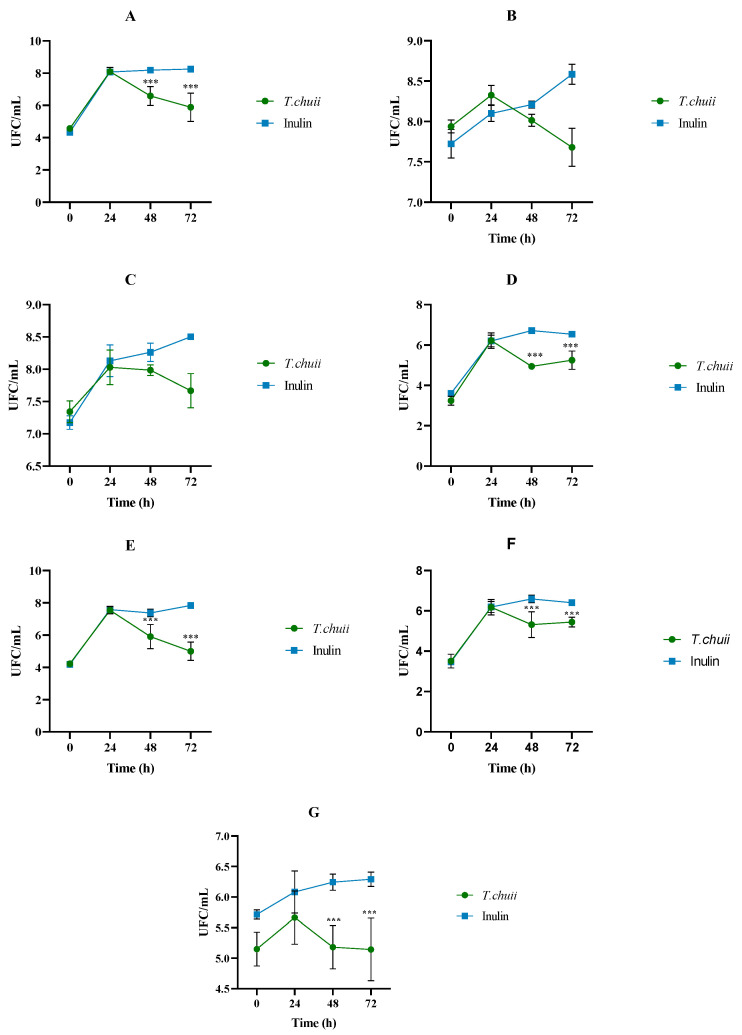
Progression of the effect of the non-absorbable fractions (NAFs) of *Tetraselmis chuii* digests and inulin on gut microbiota microbial counts (log colony-forming units (CFUs)/mL) after 0, 24, 48, and 72 h of colonic fermentation. (**A**) Total aerobes, (**B**) total anaerobes, (**C**) *Clostridium* sp., (**D**) *Staphylococcus* sp., (**E**) *Enterobacteriaceae*, (**F**) *Enterococcus* spp., (**G**) lactic acid bacteria (*n* = 3 of a pool of 5 digestions at each time). *** Differences in values were considered significant when they were higher or lower than 1 log (CFU/mL) compared to the inulin (control).

Bacterial counts in selective and general media are good indicators for a preliminary examination of the effect of the diet on the intestinal microbiota. Figure 1 depicts the mean values expressed as the log CFU/mL of total aerobes, total anaerobes, *Clostridium* sp., *Staphylococcus* sp., and *Enterobacteriaceae* for the NAF from colonic digestions at different incubation times. In the case of total aerobes (Figure 1A), a sharp increase was observed at 24 h both in the treatment with inulin (8.07 CFU/mL) and with *T. chuii* (8.10 CFU/mL). After this time, the values quantified in the inulin sample remained practically constant until the end of fermentation (8.16 CFU/mL). In comparison, in *T. chuii* digests, at 48 h, a significant decrease in the microbial populations was observed up to values of 5.89 CFU/mL (*p* < 0.0001). As for total anaerobes (Figure 1B), the microbiological behavior was very similar. In *T. chuii* samples, the microbial populations reached a maximum at 24 h (8.33 CFU/mL), decreasing over time to 7.68 CFU/mL at 72 h. For inulin, there was a gradual increase from 7.72 CFU/mL at the beginning of colonic fermentation to 8.58 CFU/mL at the end.

The counts of the most important microbial groups (*Clostridium*, *Staphylococcus* spp., *Enterobacteriaceae*, *Enterococcus*, and lactic acid bacteria, Figure 1C–G) throughout the study period were also monitored. The trends that can be seen are not very different from those shown for aerobes and anaerobes’ total microorganisms. Regardless of the type of sample (*T. chuii* or inulin), in all cases, there were strong increases in the populations at 24 h. At this point, except for lactic acid bacteria, the bacterial load did not differ between samples with inulin or *T. chuii*. Notwithstanding, thereafter, while the content in the control samples remained almost stable without major modifications, in *T. chuii* it decreased for all groups. The generalized increase in all microbial populations at 24 h after colonic fermentation would indicate an adaptation of the bacteria present to the culture medium. The decrease observed at 48 h in the samples grown in the presence of *T. chuii* (Figure 1) has no precedent in the literature for this microalga, but could be attributed to a depletion of nutrients, which would negatively affect bacterial growth. Zhou et al. [27], using extracts of a variety of microalgae, showed that not all species suppressed bacterial growth. They indicated that the inhibitory effect on microorganisms differed according to both microalgae species and extraction methods, which would affect the biomass composition in the extract, and consequently, the proliferation of bacteria. The evolution of lactic acid bacteria and *Enterococcus* spp. content during 72 h in this preliminary assay would show that the use of *T. chuii* compared to fructopolysaccharides, such as inulin, would not provide nutritional advantages. Both groups of bacteria are considered probiotics, microorganisms that, when present in adequate amounts, exert a beneficial effect on human health [28,29,30]. However, in contrast, the decrease in *Clostridium* spp. (Figure 1C), and the significant decrease in *Staphylococcus* spp. (Figure 1D) and *Enterobacteriaceae*’s (Figure 1E) relative abundances in gut microbiota would be desired from a host health perspective. These groups of microorganisms include several important foodborne pathogens. Toxins usually mediate diseases that they can develop, and their presence within the organism can lead to infections, the severity of which depends mainly on the pathological capacity or virulence of the species in question and the characteristics of the host.

### 3.2. Effects of T. chuii on the Intestinal Bacterial Populations During Colonic Fermentation: Sequencing of Bacterial 16S rRNA

**Figure 2:**
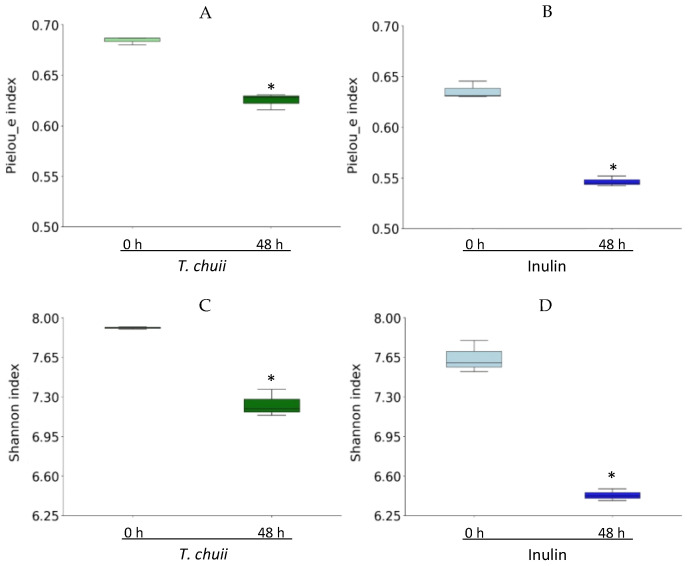
Effect of the non-absorbable fraction (NAF) from *Tetraselmis chuii* digests and inulin on α-diversity represented by Pielou’s evenness index (**A,B**), and Shannon index (**C**,**D**). The results are shown in box plots. * indicate significant differences compared to time zero.

To further investigate the impact of NAF of *T. chuii* and inulin on the gut microbiota composition, bacterial 16S rRNA was sequenced by next-generation sequencing (NGS). First, the α-diversity of each group was investigated by calculating the Pielou’s index (which evaluates the community evenness) and the Shannon index (which considers both richness and evenness) at 0 h and after 48 h. As shown in Figure 2, the analysis of these indices revealed a declining trend in overall microbial diversity after fermentation, likely influenced by the selective expansion of dominant taxa that may have competitively suppressed the growth of less abundant species. Both Pielou’s and Shannon indices were higher in the *T. chuii* group than in the inulin group after 48 h. These data show that samples incubated with NAF of *T. chuii* exhibited a more species-rich and more evenly distributed microbial community, which could indicate greater ecological stability and functional resilience.

**Figure 3:**
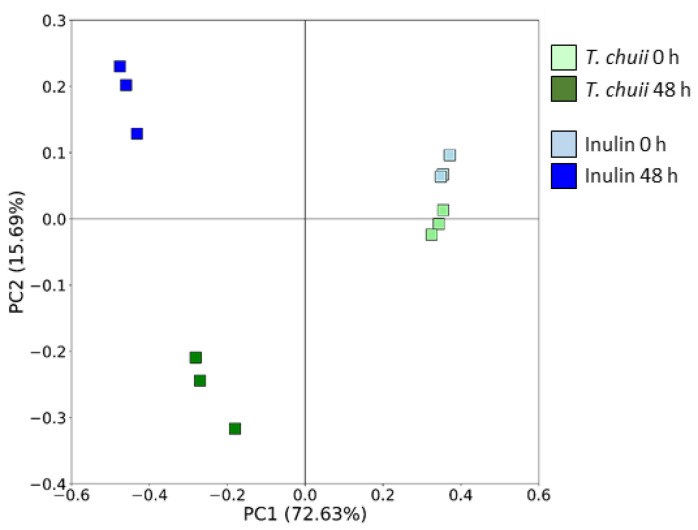
Effect of the non-absorbable fraction (NAF) from *Tetraselmis chuii* digests and inulin on β-diversity represented by a principal coordinate analysis (PCoA) plot of Bray–Curtis index dissimilarity.

Regarding the β-diversity, which compares the biodiversity among groups, it was assessed using the Bray−Curtis metric distance to calculate the distance between groups. After 48 h of fermentation, the samples of both *T. chuii* and inulin NAFs presented significant differences in the microbiota structure composition. The first two principal coordinates presented over 88% of the bacterial community variation (Figure 3).

**Figure 4:**
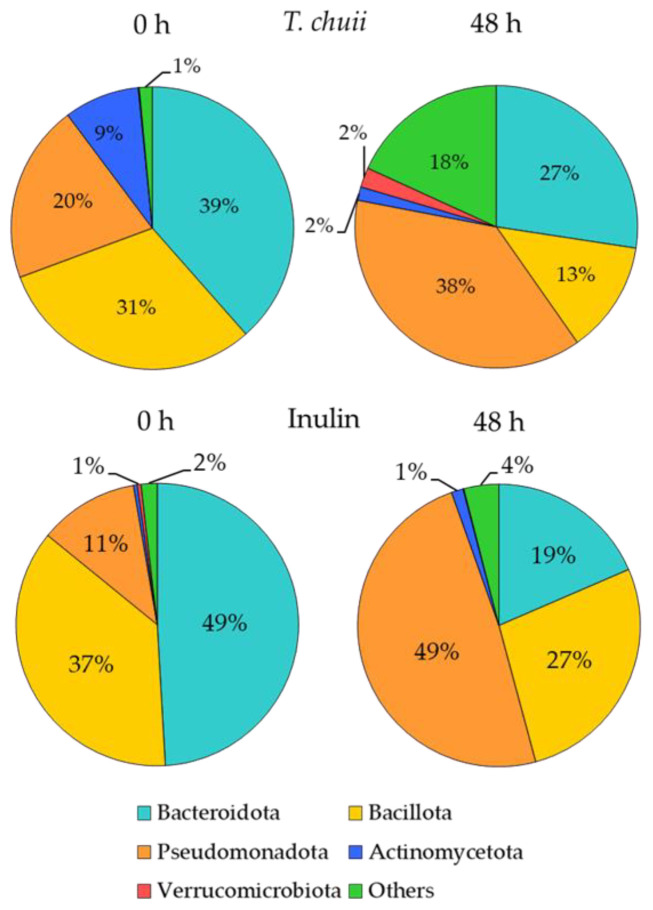
Effect of the on-absorbable fraction (NAF) from *Tetraselmis chuii* digests and inulin on fecal microbiota composition at phylum level at zero time and after 48 h. *n* = 3 (of a pool of 5 digestions).

To determine how *T. chuii* NAF affected the fecal microbiota, a taxonomic analysis was performed. Figure 4 shows the relative abundance of microbial communities in the *T. chuii* and inulin groups at the phylum level. The dominant phyla in both groups were Bacteroidota, Pseudomonadota, and Bacillota. After 48 h of fermentation, the composition of the microbiota shifted significantly in both groups. In the *T. chuii* samples, the abundance of bacteria from the Bacteroidota and Bacillota phyla decreased, whereas bacteria from the Pseudomonadota phylum, mainly composed of potentially pathogenic taxa, increased notably. Similarly, inulin reduced the abundance of bacteria belonging to Bacteroidota and Bacillota, while promoting a greater increase in Pseudomonadota compared to *T. chuii*. Interestingly, Actinomycetota and Verrucomicrobiota, two phyla often associated with health benefits, increased in the inulin and *T. chuii* groups, respectively, but decreased in the opposite group. Our results were consistent with previous reports showing that the conditions of in vitro fermentation decrease the pH, exerting a higher inhibitory effect on the growth of Bacteroidota than on Bacillota, and promoting an increment in Pseudomonadota [31,32]. Our results were consistent with previous reports showing that the conditions of in vitro fermentation decrease the pH, exerting a higher inhibitory effect on the growth of Bacteroidota than on Bacillota, and promoting an increment in Pseudomonadota [31,32]. However, since changes in the abundance of Bacteroidota and Pseudomanadota phyla differed between the *T. chuii* samples and inulin samples, our data suggest a differential effect of *T. chuii* and inulin on the gut microbiota composition.

**Figure 5:**
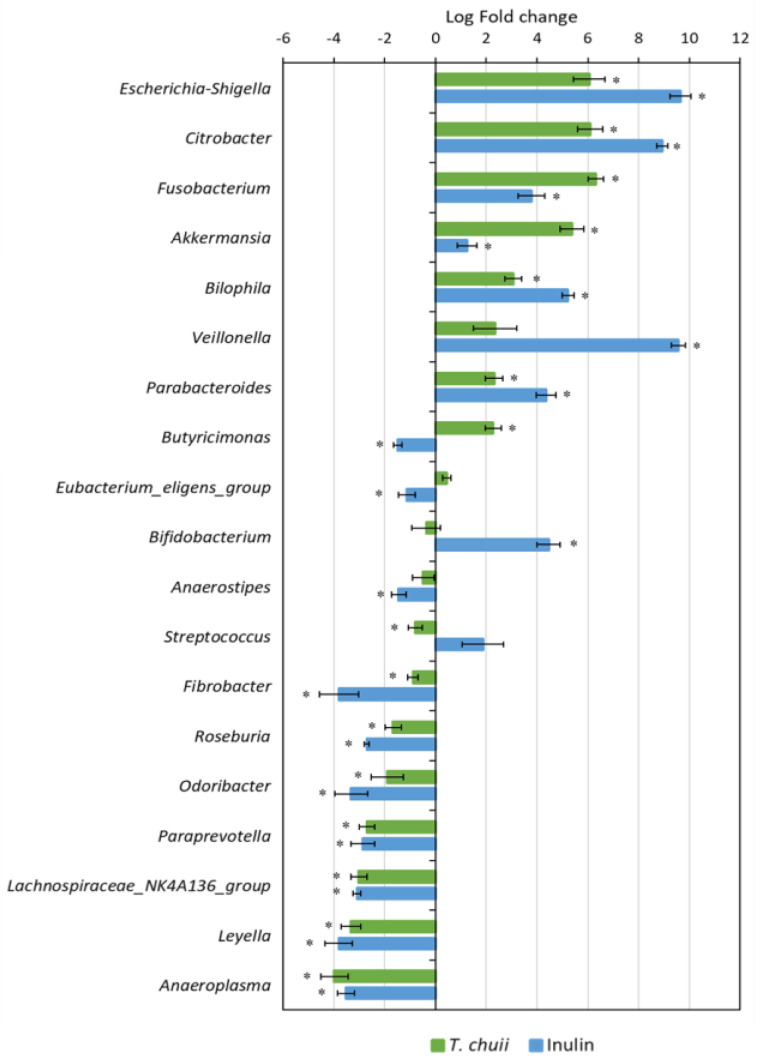
Differences in relative abundance of bacteria genera between zero time and 48 h after the fermentation with NAFs from *Tetraselmis chuii* digests and inulin. Data are represented by effect size (log-fold change) and standard error bars. *n* = 3 (of a pool of 5 digestions). * *p* < 0.05 vs. zero time.

Then, we investigated what genera could be responsible for the differences observed at the phylum level. As shown in Figure 5, the abundance of 19 kinds of main genera was found significantly changed in comparison with the beginning of the fermentation (*p* < 0.05). Compared with zero time, six genera were significantly increased (*Escherichia–Shigella*, *Citrobacter*, *Fusobacterium*, *Akkermansia*, *Bilophila*, and *Parabacteroides*), and seven decreased (*Fibrobacter*, *Roseburia*, *Odoribacter*, *Paraprevotella*, *Lachnospiraceae*_NK4A136_group, *Leyella*, and *Anaeroplasma*) in both *T. chuii* and inulin samples (Figure 5). By contrast, Butyricimonas was only significantly increased in the *T. chuii* samples; meanwhile, Bifidobacterium was only increased in the inulin samples.

**Figure 6:**
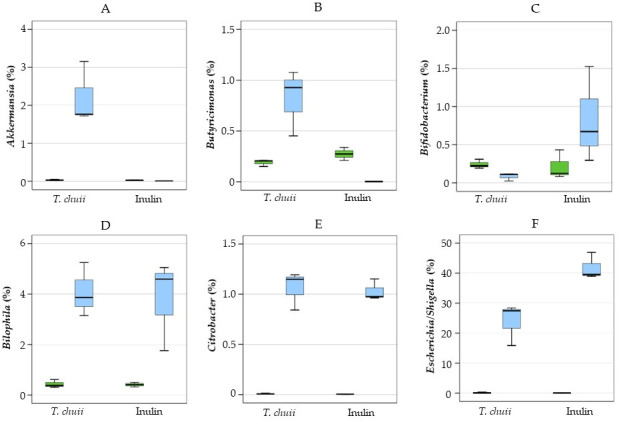
Gut microbiota composition: (**A**) Akkermansia, (**B**) Butyricimonas, (**C**) Bifidobacterium, (**D**) Bilophila, (**E**) Citrobacter, (**F**) Escherichia/Shighella at genus level of NAFs from *Tetraselmis chuii* digests and inulin at zero time and after 48 h. *n* = 3 (of a pool of 5 digestions) at each time.

In Figure 6, the percentages of the most relevant genera are shown. *T. chuii* increased the relative abundance of *Akkermansia* and *Butyricimonas*, but inulin increased *Bifidobacterium*. The genus *Akkermansia* is considered highly beneficial. It specializes in mucin degradation and is associated with weight control, insulin sensitivity, and the maintenance of intestinal barrier integrity [33,34]. *Butyricimonas* is another beneficial bacterium that produces butyrate, contributing to anti-inflammatory effects and supporting gut health [35].

The genus *Bifidobacterium* is considered highly beneficial due to its role as a key probiotic. It is associated with the production of SCFAs, particularly butyrate, which contribute to the maintenance of intestinal barrier integrity and modulation of immune responses [36]. In contrast, both *T. chuii* and inulin NAF further increased the abundance of proinflammatory bacteria such as *Bilophila*, *Citrobacter*, and, especially, *Escherichia-Shigella*. The increase in *Escherichia-Shigella* abundance induced by inulin was greater than that observed with *T. chuii*, consistent with the higher relative abundance of the phylum *Pseudomonadota*. The genera *Escherichia/Shigella* encompass both commensal and pathogenic strains. While certain *E. coli* strains are integral to gut health, other species are associated with gastrointestinal infections and inflammatory conditions [37]. *Citrobacter* are opportunistic pathogens. They can cause a range of infections, such as urinary tract infections, bloodstream infections, and meningitis, particularly in immunocompromised individuals [38]. *Bilophila* are sulfur-reducing bacteria that, under certain conditions, can contribute to intestinal inflammation and metabolic disorders [39]. Its abundance is associated with high-fat diets and conditions like metabolic syndrome [40], indicating its potential role in promoting gut inflammation.

The distinct effects of *T. chuii* and inulin NAF on gut microbiota composition could be due to the different composition of their dried matter, suggesting differential prebiotic potential. We have previously reported that the nutritional profile of *T. chuii* is characterized by significant amounts of proteins (around 32%), lipids (around 35%), and dietary fibers (25%), but low carbohydrate content (around 7%) [17,41]. In contrast, inulin consists almost entirely of carbohydrates, with a high dietary fiber content ranging from 88% to 92%, ideally suited to selectively stimulate the growth of beneficial bacteria such as *Bifidobacterium* [42].

### 3.3. Short-Chain Fatty Acid Profiles from In Vitro Human Colonic Fermentation Samples

**Figure 7:**
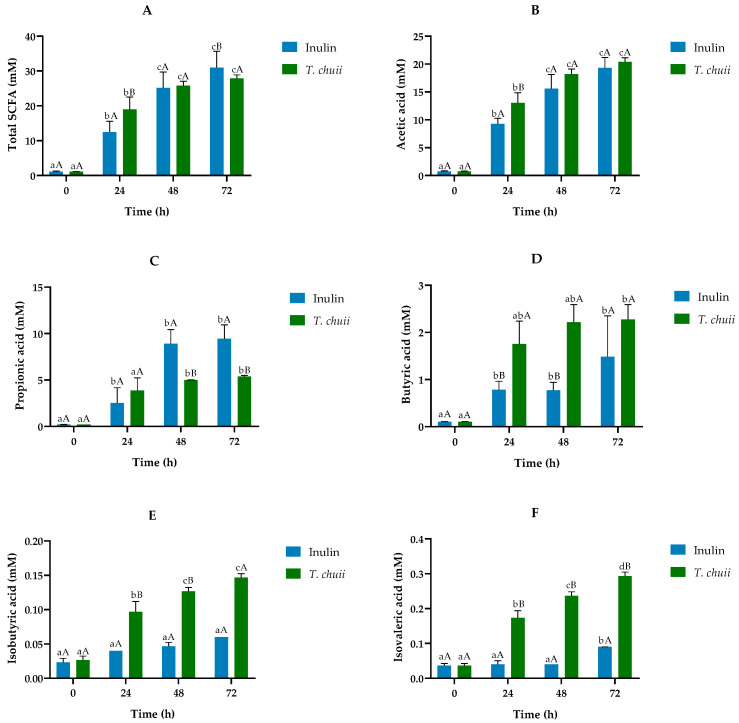
Short chain fatty acids (mMs) throughout the colonic fermentation of the non-absorbable fractions (NAFs) of *Tetraselmis chuii* digests and inulin (control) at 0, 24, 48, and 72 h. (**A**) Total SCFA; (**B**) acetic acid; (**C**) propionic acid; (**D**) butyric acid; (**E**) isobutyric acid; (**F**) isovaleric acid. *n* = 3 (of a pool of 5 digestions) at each time. Different letters indicate statistically significant differences between results, lowercase letters refer to comparisons at different fermentation times within *T. chuii* or inulin samples. In contrast, capital letters refer to comparisons at the same fermentation time between *T. chuii* and inulin samples.

The metabolic functionality of microbiota was determined by measuring the SCFA contents in the fermentation extracts. SCFAs are usually produced during the colonic fermentation process by the intestinal microbiota. Figure 7 shows the evolution of the concentration of total and individual SCFA, both major (acetic, propionic, and butyric) and minor (isobutyric and isovaleric), during the fermentation process for *T. chuii* and inulin digests. The presence of the total SCFA in the microalgae digest increased the total levels up to 30 mM, not differing significantly throughout the experimental period from the samples incubated with the standard fructopolysaccharide. This fact would support the role of *T. chuii* as a prebiotic ingredient in the diet. Essential for gut integrity, microbial SCFAs regulate luminal pH, promote mucus production, provide epithelial cell fuel, and affect mucosal immune function. Furthermore, they directly modulate host metabolic health through tissue-specific mechanisms related to appetite regulation, energy expenditure, glucose homeostasis, and immunomodulation. All the individual major SCFAs experienced a strong increase throughout the incubation period with *T. chuii* (Figure 7A). These results agreed with similar previous studies performed with the biomass extracts of other microalgae such as *Chlorella vulgaris*, *Spirulina platensis*, or *Phaeodactylum tricornutum*, among others [15,27,43].

When the majority of individual SCFA values in *T. chuii* and inulin were compared, the trends were variable. The evolution of acetic acid contents was very similar, with hardly any differences between the two substrates (Figure 7B). Propionic acid was higher in samples with inulin after 48 h and 72 h (Figure 7C). In contrast, butyric acid was more abundant in *T. chuii* throughout the incubation period, and significantly so at 48 h (Figure 7D). These data agreed with the increase in *Akkermansia* and *Butyricimonas* (Figure 6), genera associated with mucosal integrity and butyrate production, respectively, and is quite relevant because butyrate is considered an important energy source for colonocytes, responsible for functions linked to the gut microbiota and physiology, maintaining cell growth and differentiation [15].

Furthermore, although butyric acid itself is an important metabolite, their producers could be much more relevant as they actively control the gut microbiome via various anti-microbial and anti-inflammatory molecules [19].

Among the minority SCFA, the presence of isobutyric and isovaleric (Figure 7E,F), both branched-chain fatty acids (BCFAs), stands out quantitatively. Their presence is, at least, one order of magnitude lower than that of butyric acid, reaching their maximum values, 0.15 (isobutyric) and 0.30 mM (isovaleric), at the end of colonic fermentation. These values were measured in samples containing *T. chuii*, since in the inulin digest, their concentrations were very low. Gut microbiota, by branched-chain amino acid fermentation, can produce low levels of these BCFA [44]. Isobutyrate could function as a carbon source for energy in colonocytes under conditions of low butyrate availability [45], and isovaleric acid can act directly on the colonic smooth muscle, causing relaxation [46]. Both BCFAs have effects on adipocyte lipid and glucose metabolism that could contribute to improved insulin sensitivity in individuals with disturbed metabolism [47]. However, little is still known about the possible molecular mechanisms of action and their interactions with microbiota and the gut of these fatty acids.

### 3.4. Evolution of the Proteolytic Activity

Table 1 shows the evolution of the protein and ammonium contents during the colonic fermentation of samples incubated with *T. chuii* or inulin digests. Protein levels decreased gradually throughout the study period, and, at 48 h, more than one-third had already been metabolized. Simultaneously, from 24 h after the onset of colonic fermentation, the ammonium content increased exponentially by more than six, to reach values above 300 mg/L at the end of the study. Furthermore, the contents of this ion were significantly higher than those measured in samples incubated with inulin during the experimental period.

Overall, dried *T. chuii* is considered a good source of proteins with a desirable amino acid profile [26,48,49]. This species of microalgae has protein levels of approximately dry matter 30%, mainly depending on the growing environmental factors [13]. Concerning ammonium ions, information on the evolution of its content during colonic fermentation in microalgae extracts remains very scarce [17]. The increase in ammonia detected after *T. chuii* digest treatment could be attributed to its protein composition, as ammonia is the principal metabolite resulting from intestinal bacterial protein fermentation. These ions result from amino acids’ deamination by intestinal microbiota [50]. Although most dietary proteins are digested and absorbed in the small intestine, a load of unabsorbed proteins and peptides would reach the colon, where they could serve as a substrate for fermentation by the resident microorganisms. In the colon, such protein substrates would yield amino acids, which, in turn, could be subjected to different secondary fermentation pathways by gut bacteria. Ammonium, together with CO_2_ and organic acids, would be the primary products of such amino acid fermentation [17]. Therefore, the significant presence of ammonium during the colonic fermentation of *T. chuii* extract measured in the present research is important evidence of the digestibility of this microalgae extract.

## 4. Conclusions

The use of *T. chuii* microalgae may have an important potential as a functional ingredient in human feeding. The passage of this microalga through the gastrointestinal tract, combining simulated gastrointestinal digestion with in vitro colonic fermentation, modified the human colonic microbiota composition and its metabolic functionality. The presence of *T. chuii* during the digestive process favored the proliferation of different bacterial groups, some of which, such as *Akkermansia* and *Butyricimonas,* could be beneficial to health. The increase in SCFA, particularly butyric acid, generated in the samples incubated with *T. chuii* digest would support the role of this microalgae as a prebiotic ingredient. Although this study represents a preliminary step in evaluating the impact of in vitro *T. chuii* digests on gut health, the findings highlight the potential of this microalga and require more in-depth investigation. Further research, including dynamic digestion models and in vivo trials, is encouraged to confirm its efficacy and to better understand interindividual variability in response to *T. chuii* intake, which is essential for its development as a scientifically validated nutraceutical for the modulation of gut microbiota.

## Figures and Tables

**Table 1:** Protein content (µg/mL) and ammonium production (mg/L) throughout the colonic fermentation of the non-absorbable fractions (NAFs) of *Tetraselmis chuii* digests and inulin (control) at 0, 24, 48, and 72 h.

		Protein (µg/mL)	Ammonium (mg/L)
*Tetraselmis chuii*	0 h	3056 ± 171 ^bB^	31.92 ± 2.18 ^cA^
	24 h	1934 ± 74 ^aB^	269.20 ± 25.38 ^bA^
	48 h	1922 ± 106 ^aA^	309.02 ± 16.83 ^abA^
	72 h	2003 ± 39 ^aA^	324.03 ± 12.48 ^aA^
Inulin	0 h	3362 ± 215 ^cA^	33.88 ± 7.52 ^dA^
	24 h	2490 ± 103 ^bA^	105.31 ± 9.94 ^cB^
	48 h	1930 ± 85 ^aA^	131.56 ± 3.10 ^bB^
	72 h	2062 ± 108 ^aA^	200.16 ± 11.54 ^aB^

Different letters indicate statistically significant differences between results, lowercase letters refer to comparisons at different fermentation times within *T. chuii* or inulin samples, while capital letters refer to comparisons at the same fermentation time between *T. chuii* and inulin samples. *n* = 3 (of a pool of 5 digestions) at each time.

## Data Availability

The original contributions presented in this study are included in the article. Additionally, raw data and metadata have been deposited with the BioProject database at the National Center for Biotechnology Information (NCBI), accessible at http://www.ncbi.nlm.nih.gov/bioproject/1255441. Further inquiries can be directed to the corresponding author(s).
